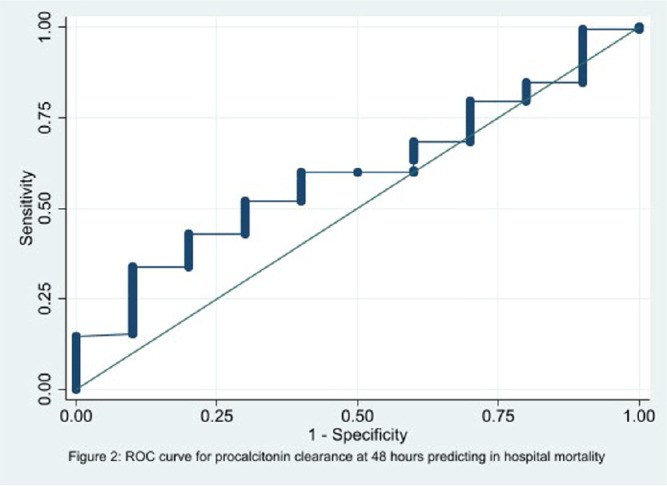# Does Serial Procalcitonin Monitoring predict Clinical Outcomes in Children with Sepsis? A diagnostic stewardship study

**DOI:** 10.1017/ash.2024.216

**Published:** 2024-09-16

**Authors:** Beenish Rubbab, Zachary Most

**Affiliations:** UTSW; Children’s Health System of Texas

## Abstract

**Background:** In the management of children with sepsis, inflammatory markers are often obtained upon admission and repeated frequently. It is unclear if serial monitoring of procalcitonin is useful for predicting patient outcomes. The focus of our study is to identify if the trend of procalcitonin levels was predictive of the clinical outcomes in children with sepsis. **Methods:** We performed a retrospective diagnostic study to evaluate the association between change in procalcitonin levels and clinical outcomes. Encounters for children 1 to 8 years old with a sepsis ICD 10 diagnosis code and meeting the clinical sepsis criteria from May 2020 to May 2022 at one quaternary care pediatric hospital were included. Encounters with fewer than two procalcitonin values and children with autoimmune diseases, trauma, new onset malignancy, and fungal infections were excluded. Procalcitonin clearance at 48 hours (CL-PCT48) was defined as the difference in procalcitonin values drawn on admission and at 48 hours divided by initial procalcitonin value. The primary outcome was good early clinical response, defined as composite measure of temperature, hemodynamic stability, supplemental oxygen requirement, and need for renal replacement therapy at 120 hours of admission. All-cause in-hospital mortality was a secondary outcome. ROC analysis was performed to measure the correlation of CL-PCT48 and initial procalcitonin value (PCT0) with the outcome. **Result:** There were 320 unique encounters for children who met the clinical criteria of sepsis. The median number of procalcitonin measurements was 4 (Range 2 – 111). Of these encounters, 187 had procalcitonin measurements done at eligible times. The mean age of the study participants was 9 years and 8 months, 103 (55%) were male, and the majority (54%) were Caucasian. Fifty-seven (30%) individuals had bacterial growth from a culture from sterile body fluid or urine. 78 (41.7%) individuals had good early clinical response and 177 (94.7%) survived to hospital discharge. There was no correlation identified between CL-PCT48 and good early clinical response (area under ROC curve [AUC] = 0.57, 95% CI 0.48-0.65, Figure 1) or mortality (AUC = 0.60, 95% CI 0.43-0.76, Figure 2). There was also no correlation between PCT0 and good early clinical response (AUC = 0.47, 95% CI 0.39-0.56) or and mortality (AUC = 0.50, 95% CI 0.29-0.72). **Conclusion:** Procalcitonin clearance at 48 hours after admission did not predict early clinical response in children with sepsis.

**Figure:**
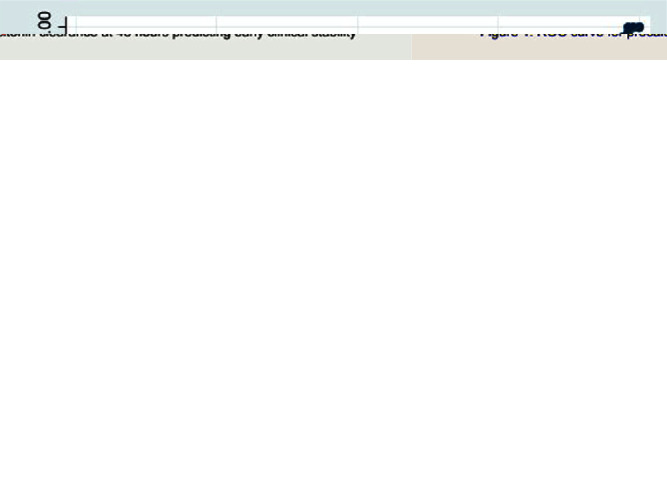

**Figure:**